# Protein and Peptide Drug Delivery: Oral Approaches

**DOI:** 10.4103/0250-474X.42967

**Published:** 2008

**Authors:** Jessy Shaji, V. Patole

**Affiliations:** Department of Pharmaceutical Sciences, Prin. K. M. Kundnani College of Pharmacy, Cuffe Parade, Mumbai-400 005, India

**Keywords:** Therapeutic proteins and peptides, oral delivery, formulation vehicles, absorption enhancers, enzyme inhibitors, mucoadhesive polymeric system

## Abstract

Till recent, injections remained the most common means for administering therapeutic proteins and peptides because of their poor oral bioavailability. However, oral route would be preferred to any other route because of its high levels of patient acceptance and long term compliance, which increases the therapeutic value of the drug. Designing and formulating a polypeptide drug delivery through the gastro intestinal tract has been a persistent challenge because of their unfavorable physicochemical properties, which includes enzymatic degradation, poor membrane permeability and large molecular size. The main challenge is to improve the oral bioavailability from less than 1% to at least 30-50%. Consequently, efforts have intensified over the past few decades, where every oral dosage form used for the conventional small molecule drugs has been used to explore oral protein and peptide delivery. Various strategies currently under investigation include chemical modification, formulation vehicles and use of enzyme inhibitors, absorption enhancers and mucoadhesive polymers. This review summarizes different pharmaceutical approaches which overcome various physiological barriers that help to improve oral bioavailability that ultimately achieve formulation goals for oral delivery.

Due to rapid progress in biotechnology, as well as gene technology, the industry is capable of producing a large number of potential therapeutic peptides and proteins in commercial quantities. Endogenous proteins and peptides play an important role in the regulation and integration of life processes and act with high specificity and potency^1^. For example, in the form of enzymes, hormones, antibodies and globulins, they catalyze, regulate and protect the body chemistry, while in the form of haemoglobin, myoglobin and various lipoproteins, they affect the transport of oxygen and other chemical substances within the body. In the form of skin, hair, cartilage and muscles, proteins hold together, protect and provide structure to the body of a multicellular organism^2^.

The increasing importance of proteins and peptides can be attributed to three main developments. First, improved analytical methods have promoted the discovery of numerous hormones and peptides that have found applications as biopharmaceuticals. Second, molecular biology and genetic engineering have enabled the large-scale production of polypeptides previously available only in small quantities. Lastly, there is a better understanding of the role of regulatory proteins/peptides in the pathophysiology of human diseases^2^,^3^. Simultaneously, pharmaceutical companies around the world have endeavored to develop the processes for producing therapeutically active entities at commercial scales.

Till recently, injections (i.e. intravenous, intramuscular or subcutaneous route) remain the most common means for administering these protein and peptide drugs. Patient compliance with drug administration regimens by any of these parenteral routes is generally poor and severely restricts the therapeutic value of the drug, particularly for disease such as diabetes^1^. Among the alternate routes that have been tried with varying degrees of success are the oral, buccal^4^, intranasal^5^, pulmonary^6^, transdermal^7^, ocular^8^ and rectal^9^. Among these, oral route remains the most convenient way of delivering drugs. Oral administration presents a series of attractive advantages towards other drug delivery. These advantages are particularly relevant for the treatment of pediatric patients and include the avoidance of pain and discomfort associated with injections and the elimination of possible infections caused by inappropriate use or reuse of needles. Moreover, oral formulations are less expensive to produce, because they do not need to be manufactured under sterile conditions^10^. In addition, a growing body of data suggests that for certain polypeptides such as insulin; the oral delivery route is more physiological^11^.

Designing oral peptide and protein delivery systems has been a persistent challenge to pharmaceutical scientists because of their several unfavorable physicochemical properties including large molecular size, susceptibility to enzymatic degradation, short plasma half-life, ion permeability, immunogenicity, and the tendency to undergo aggregation, adsorption, and denaturation^12^,^13^. Consequently, the absolute oral bioavailability levels of most peptides and proteins are less than 1%. The challenge here is to improve the oral bioavailability from less than 1% to atleast 30-50%^14^.

Designing and formulating a protein and peptide drug for delivery though GI tract requires a multitude of strategies. The dosage form must initially stabilize the drug making it easy to take orally^4^. It must then protect the drug from the extreme acidity and action of pepsin in the stomach. In the intestine, the drug should be protected from the plethora of enzymes that are present in the intestinal lumen. In addition, the formulation must facilitate both aqueous solubility at near-neutral pH and lipid layer penetration in order for the protein to cross the intestinal membrane and then basal membrane for entry into the bloodstream.

The purpose of this article is to review the general approaches that have been studied for improving oral protein and peptide bioavailability by overcoming various physiological barriers associated with therapeutic proteins and peptides.

## PHARMACEUTICAL APPROACHES

Table 1 lists several pharmaceutical approaches that are available for maximizing oral protein and peptide absorption.

**TABLE 1:** VARIOUS PHARMACEUTICAL APPROACHES AND THEIR OUTCOMES

Approaches	Outcomes
Chemical modification	
a) Amino acid odification	Improves enzymatic stability.
b) Hydrophobization	Improve membrane penetration
Use of enzyme inhibitors	Resist degradation by enzymes present in stomach and intestine
Use of absorption enhancers	Increases membrane permeability
Formulation vehicles	
a) Emulsions	Protects drug from acid and luminal proteases in the GIT. Enhance permeation through intestinal mucosa
b) Microspheres	Prevents proteolytic degradation in stomach and upper portion of small intestine. Restricts release of drug to favorable area of GIT
c) Nanoparticles	Prevent enzymatic degradation. Increases intestinal epithelial absorption
d) Liposomes	Improves physical stability. Increases membrane permeability.
Mucoadhesive polymeric system	Achieve site-specific drug delivery. Improves membrane permeation.

### Chemical modification:

A chemical modification of peptide and protein drugs improves their enzymatic stability and/or membrane penetration of peptides and proteins. It can also be used for minimizing immunogenicity. Protein modification can be done either by direct modification of exposed side-chain amino acid groups of proteins or through the carbohydrate part of glycoproteins and glycoenzymes^15^.

Modifications of individual amino acids combined with the substitution of one more L-amino acid with D-amino acids can significantly alter physiological properties. This was demonstrated by vasopressin analogs 1-deamino-8-D-arginine vasopressin (DDAVP) and [Val4, D-Arg8], arginine-vasopressin (dVDAVP), hereafter called desmopressin and deaminovasopressin, respectively. While the former involves deamination of the first amino acid and replacement of the last L-arginine with D-arginine, the latter also has the fourth amino acid changed to valine. While the natural vasopressin is orally active in the water-loaded rat at large doses, desmopressin is twice as active at the 75^th^ fraction of the dose, which is attributed to enhanced membrane permeation and enzymatic stability. Desmopressin absorption was shown to be passive and by the paracellular route across the rat jejunum and site dependent in rabbits. Whether the chemical modification alters the transport pathway, however, remains to be unknown^15^.

Increasing the hydrophobicity of a peptide or protein by surface modification using lipophilic moieties may be of particular benefit to transcellular passive or active absorption by membrane penetration or attachment, respectively; or it may simply aid in the increased stability of the protein.

Nobex corporation has developed a proprietary insulin compound modified with small polymers (chemical name of the Nobex insulin is hexyl-insulin-monoconjugate 2 or “HIM2”), in which a single amphiphilic oligomer is covalently linked to the free amino group on the Lys-β29 residue of recombinant human insulin via an amide bond^16^, that is intended, on delivery by mouth, to resist degradation by enzymes of the stomach and intestine and to be efficiently absorbed into the bloodstream. It is believed that once delivered by mouth to the intestine and into the bloodstream, Nobex oral insulin can follow the same pathway as insulin released by the pancreas, into a blood vessel called the portal vein and then directly to the liver. Since the liver is a significant participant in the control of blood glucose, it is believed that successfully activating the liver with oral insulin may provide a mechanism to potentially reestablish normal glucose control in the diabetic patient and turn on a number of metabolic activities that can help mitigate complications of diabetes^17^.

Another example of hydrophobization to increase lipophilicity of insulin is palmitoylation. Insulin was conjugated to 1,3-dilpalmitoylglycerol at the free amino groups of glycine, phenylalanine, and lysine to form mono and dipalmitoyl insulin^18^. This facilitated the transfer of insulin across the mucosal membranes of the large intestine and improved its stability against intestinal enzymatic degradation. To decrease binding to albumin, Brader *et al*.^19^ recently synthesized octanoyl-N-Lysβ-29, co-crystallized with human insulin, and determined pharmacokinetic and insulin release profiles after subcutaneous injection in beagle dogs. However, these derivatives were not very effective after oral administration.

### Enzyme inhibitors:

The choice of protease inhibitors will depend on the structure of these therapeutic drugs, and the information on the specificity of proteases is essential to guarantee the stability of the drugs in the GI tract^20^. The quantity of co-administered inhibitor(s) is essential for the intestinal permeability of a peptide or protein drug.

For example, enzyme degradation of insulin is known to be mediated by the serine proteases trypsin, α-chymotrypsin and thiol metalloproteinase insulin degrading enzymes. The stability of insulin has been evaluated in the presence of excipients that inhibit these enzymes. Representative inhibitors of trypsin and α-chymotrypsin include pancreatic inhibitor and soybean trypsin inhibitor, FK-448, Camostat mesylate and aprotinin. Inhibitors of insulin degrading enzymes include 1,10-phenanthroline, p-chloromeribenzoate and bacitracin reported the use of a combination of an enhancer, sodium cholate and a protease inhibitor to achieve a 10% increase in rat intestinal insulin absorption^1^.

Thiomers are promising candidates within as enzyme inhibitors. Hutton *et al*.^21^ first reported the inhibitory properties of poly (acrylates) on intestinal proteases. They found a strong reduction of albumin degradation by a mixture of proteases in the presence of carbopol 934P. A subsequent study by Lueben *et al*.^22^ showed that polycarbophil and carbopol 934P were potent inhibitors of the proteolytic enzymes trypsin, α-chymotrypsin and carboxypeptidase A. As a result of the covalent attachment of cysteine to polycarbophil, the inhibitory effect of the polymer towards carboxypeptidase A, carboxypeptidase B and chymotrypsin could be significantly improved. This polycarbophil-cysteine conjugate also had a significantly greater inhibitory activity than unmodified polycarbophil on the activity of isolated aminopeptidase N and aminopeptidase N present on intact intestinal mucosa^23^.

Another approach to enzyme inhibition is to manipulate the pH to inactivate local digestive enzymes. A sufficient amount of a pH-lowering buffer that lowers local intestinal pH to values below 4.5 can deactivate trypsin, chymotrypsin and elastase^1^.

### Absorption enhancers:

In order for therapeutic agents to exert their pharmacological effects, they have to cross from the biological membranes into the systemic circulation and reach the site of action. Absorption enhancers are the formulation components that temporarily disrupt the intestinal barrier to improve the permeation of these drugs. Ideally, the action of absorption enhancers should be immediate and should coincide with the presence of the drug at the absorption site.

Numerous classes of compounds with diverse chemical properties, including detergents, surfactants, bile salts, Ca^2+^ chelating agents, fatty acids, medium chain glycerides, acyl carnitine, alkanoyl cholines, N-acetylated α-amino acids, N-acetylated non-α-amino acids, chitosans, mucoadhesive polymers, and phospholipids have been reported to enhance the intestinal absorption of large polypeptide drugs^24^,^25^.

Many of these absorption enhancers act as detergents / surfactants to increase the transcellular transport of drugs by disrupting the structure of the lipid bilayer rendering the cell membrane more permeable and/or by increasing the solubility of insoluble drugs^26^. The chelators are believe to exert their action by complex formation with calcium ions, thus rupturing the tight junctions (TJs) and facilitate paracellular transport of hydrophilic drugs. However, permeation enhancers often induce toxic side effects, for e.g.-Ca^2+^ depletion induces global changes in the cells, including disruption of actin filaments, disruption of adherent junctions, and diminished cell adhesion^27^. Reports about some enhancers, including fatty acid sodium caprate and long chain acyl carnitines, have been shown to improve absorption without obvious harmful effects to the intestinal mucosa^28^. But based on various studies^29^–^31^, it would appear that a transient opening of TJs would seem less damaging than disruption of cell membrane structure. Several studies on sodium dodecyl sulfate, sodium caprate, and long-chain acylcarnitines shows increased permeability through the paracellular pathways^28^. Tomita *et al*.^32^ and Lindmark *et al*.^33^ proposed that the mechanism of paracellular transport enhancement by sodium caprate was via phospholipase C activation and upregulation of intracellular Ca^2+^, leading to contraction of calmodulin dependent actin-myosin filaments and opening of TJs. Dodecylphosphocholine and quillaja saponin, dipotassium glycyrrhizinate, 18β-glycyrrhetinic acid, sodium caprate, and taurine also increases the permeability of hydrophilic compounds across Caco-2 cells^26^.

Among the recent absorption enhancers displaying this principle and exhibiting the safest and most effective promising results in enhancing drug delivery is Zonula Occludens toxin or Zot. Zot is a single polypeptide chain of 44.8 kDa, 399 amino acids in length, with a predicted pI of 8.5, of bacteriophage origin, present in toxigenic stains of *V. cholerae* with the ability to reversibly alter intestinal epithelial TJs, allowing the passage of macromolecules through mucosal barriers. Zot possess multiple domains that allow a dual function as a morphogenetic phage protein and as an enterotoxin. After cleavage at amino acid residue 287, a carboxyl terminal fragment of 12 kDa is excreted, that is probably responsible for the biological effect of the toxin^10^. The mechanism of action of ZOT has been constructed as protein kinase C-dependent actin reorganization through interaction with a specific receptor, whose surface expression on various cells may differ because the action of ZOT is not uniform throughout the GI tract^34^.

*In vitro* experiments in the rabbit ileum demonstrated that Zot reversibly increased intestinal absorption of insulin (MW 5733 Da) by 72% and immunoglobulin G (140-160 kDa) by 52% in a time dependent manner They further observed an encouraging 10-fold increase in insulin absorption in both rabbit jejunum and ileum *in vivo* with ZOT^34^. Karyekar *et al*. has recently reported that Zot increases the permeability of molecular weight markers (sucrose, inulin) and chemotherapeutic agents (paclitaxel and doxorubicin) across the bovine brain microvessel endothelial cells in a reversible and concentration dependant manner and without affecting the transcellular pathway as indicated by the unaltered transport of propranolol in the presence of Zot^35^. Extensive *in vivo* and *in vitro* studies have identified Zot receptors in the small intestine, the nasal epithelium, the heart and the brain endothelium^10^. Moreover, toxicity studies have shown that Zot and its biologically active fragment ΔG do not compromise cell viability or cause membrane toxicity as compared to other absorption enhancers^10^.

Another recently developed option for the use of absorption enhancers is to co-administer protein and peptide drugs with concentrated solutions of so-called “carrier” molecules^27^,^36^–^37^. Emisphere Technologies^38^ has created a series of “transport carriers”, designed to form a complex with the polypeptide, thereby altering the structure of the polypeptide to a ‘transportable’ conformation. These molecules promote protein and peptide drug absorption. The mechanism of action of these agents is still not clear, and efforts are being made to explore the same. Leone-Bay^27^,^36^–^37^ suggested that enhanced drug permeation across the GI tract is neither due to alteration in membrane structure (i.e., mucosal damage) nor a result of direct inhibition of degradation. Based on the structure-activity relationships, these authors concluded that more lipophilic compounds (i.e., high log P values) had better ability to promote protein (rhGH, sCT) absorption^39^. They suggested that these delivery agents cause temporary stabilization of partially unfolded conformations of proteins, exposing their hydrophobic side chains. The altered lipid solubility permits them to gain access to pores of integral membrane transporter, and thus they are more absorbable through lipid bilayers^40^. Wu and Robinson used Caco-2 cell monolayers to show that interaction of rhGH with 4-(4-(2-hydroxybenzoyl) aminophenyl) butyric acid (IX) and N- (8-(2-hydroxybenzoyl) aminocaprylate (XI) makes the protein a better substrate for P-glycoprotein, thereby suggesting that the interaction causes the protein to be more lipophilic^41^.

Kotze *et al*. have evaluated the transport enhancing effects of two chitosan salts, chitosan hydrochloride and chitosan glutamate (1.5% w/v), and the partially quaternized chitosan derivative, *N*-trimethyl chitosan chloride (TMC) (1.5 and 2.5% w/v), *in vitro* in Caco-2 cell monolayers. The transport of the peptide drugs buserelin, 9-desglycinamide, 8-arginine vasopressin (DGAVP) and insulin was followed for 4 h at pH values between 4.40 and 6.20. They observed that all the chitosans (1.5%) were able to increase the transport of the peptide drugs significantly in the following order: chitosan hydrochloride>chitosan glutamate>TMC. Because of quaternary structure of TMC, it is better soluble than the chitosan salts and further increases peptide transport at higher concentrations (2.5%) of this polymer. The increases in peptide drug transport are in agreement with a lowering of the transepithelial electrical resistance (TEER) measured in the cell monolayers. No deleterious effect to the cell monolayers could be detected with the trypan blue exclusion technique. It is concluded from this study that chitosans are potent absorption enhancers, and that the charge, charge density and the structural features of chitosan salts and *N*-trimethyl chitosan chloride are important factors determining their potential use as absorption enhancers for peptide drugs^42^.

### Formulation vehicles:

A primary objective of oral delivery systems is to protect protein and peptide drugs from acid and luminal proteases in the GIT. To overcome these barriers, several formulation strategies are being investigated. Here, we discuss the use of enteric-coated dry emulsions, microspheres, liposomes and nanoparticles for oral delivery of peptides and proteins.

Emulsions protect drug from chemical and enzymatic breakdown in the intestinal lumen. Drug absorption enhancement is dependent on the type of emulsifying agent, particle size of the dispersed phase, pH, solubility of drug, type of lipid phase used etc. the lipid phase of microemulsions is composed of medium chain fatty acids triglycerides increasing the bioavailability of muramyl dipeptides analog^1^.

Torisaka *et al*. have recently prepared a new type of oral dosage form of insulin, S/O/W emulsions, in which a surfactant-insulin complex is dispersed into the oil phase^43^. This novel insulin formulation was designed to alleviate the previously mentioned two barriers: the solubilization into the oil phase can avoid degradation of protein and the noncovalent coating of insulin molecules with a lipophilic surfactant making it possible to enhance permeation through the intestinal mucosa without introducing a new chemical entity. The potential of the S/O/W emulsion was validated by hypoglycemic activity over several hours after oral administration to diabetic rats. However, a critical drawback of this formulation was physical-chemical instability in long-term storage and the requirement for storage at low temperatures^44^. To overcome this drawback, it is formulated into dry emulsion. Dry emulsion formulations are typically prepared from O/W emulsions containing a soluble or an insoluble solid carrier in the aqueous phase by spray drying^45^–^47^, lyophilization^48^ or evaporation^49^. Dry emulsions are regarded as lipid-based powder formations from which an O/W emulsion can be reconstituted. From a pharmaceutical point of view, they are attractive due to their physical strength and ease of administration as capsules and tablets. In this study, Eiichi Torisaka *et al*. have developed a unique dry emulsion formulation in which the surfactant-insulin complex was entrapped in the oil phase of the solid formulation. Using a pH-responsive polymer, HPMCP, the dry emulsion was enteric-coated^44^. The release behavior of encapsulated insulin was found to be responsive to external pH and the presence of lipase under the simulated GI conditions. Based on the results obtained in this study and the fact that any water-soluble drug can be complexed with surfactants^43^, the new solid emulsion formulations could be extensively applicable to oral delivery of pharmaceutical peptides and proteins^44^.

The influence of pH variability through the stomach to the intestine on the oral bioavailability of peptide and protein drugs may be overcome by protecting them from proteolytic degradation in the stomach and upper portion of the small intestine using pH-responsive microspheres as oral delivery vehicles. Lowman *et al*.^50^, loaded insulin into polymeric microspheres of poly (methacrylic-g-ethylene glycol) and observed oral bioavailability in healthy and diabetic rats. In the acidic environment of the stomach, the microspheres were unswollen as a result of the formation of intermolecular polymer complexes. The insulin remained in the microspheres and was protected from proteolytic degradation. While in the basic and neutral environments of the intestine, the complexes dissociated which resulted in rapid microspheres swelling and insulin release. Within 2 h of administration of the insulin-containing polymers, strong dose-dependent hypoglycemic effects were observed in both healthy and diabetic rats^50^. Numerous pH-sensitive polymers have been investigated for a range of applications^51^,^52^. These microspheres restrict the release of proteins to favorable area of GIT.

Recently, nanoparticles as particulate carriers are used to deliver protein and peptide drugs orally. It is stated that particles in the nanosize range are absorbed intact by the intestinal epithelium, especially, through peyer’s patches and travel to sites such as the liver, the spleen and other tissues^53^. The proteins and peptides encapsulated in the nanoparticles are less sensitive to enzyme degradation through their association with polymers^1^. It is demonstrated that protein and peptide encapsulated in nanoparticles have better absorption through GI tract as compared to their native counterpart. The factors affecting uptake include the particle size of particulate, the surface charge of the particles, the influence of surface ligands and the dynamic nature of particle interaction in the gut^1^.

Behrens^54^ studied the interaction of nanoparticles consisting of hydrophobic polystyrene, bioadhesive chitosans and (PLA-PEG) with two human intestinal cell lines and compared the *in vivo* uptake in rats. After intraduodenal administration of chitosans nanoparticles in rats, particles were detected in both epithelial cells and peyer’s patches. In one example, insulin was encapsulated in nanospheres using phase inversion nanoencapsulation. The insulin released over a period of appoximately 6 h, was shown to be orally active, and had 11.4% of the efficacy of intraperitoneally delivered insulin^55^.

One problem using nanoparticles is the erratic nature of nanoparticles absorption. For example, proportion of intact particles reaching systemic circulation was estimated to be generally below 5%.

Liposomes are prone to the combined degrading effects of the acidic pH of the stomach, bile salts and pancreatic lipase upon oral administration. There are several reports on the intact liposomal uptake by cells in *in vitro* and *in situ* experiments^56^–^58^. The results are, however, not convincing for the oral delivery of protein with a liposomal system. Attempts have been made to improve the stability of liposomes either by incorporating polymers at the liposome surface, or by using GI-resistant lipids^1^.

*In vitro* release of insulin, a model peptide, from liposomes in the bile salts solution was markedly reduced by coating the surface with the sugar chain portion of mucin or polyethylene glycol. Encapsulation of insulin with the sugar chain portion of mucin and that of polyethylene glycol completely suppressed the degradation of insulin in the intestinal fluid, whereas uncoated liposomes suppressed it only partially. These results demonstrated that surface coating of liposomes with PEG or mucin gained resistance against digestion by bile salts and increased the stability in the GI tract. When insulin was orally administered to rats as a solution or non-charged liposome, no hypoglycemic effect was observed. Administration of insulin encapsulated in positively charged liposome caused the rapid decrease in the plasma glucose level that recovered to the control level within 3 h. In contrast, PEG containing liposomes and mucin containing liposomes caused a gradual decrease in the glucose level after administration. The hypoglycemic effect by PEG-Liposome lasted for much longer duration than that of uncoated liposomes. The slow release of insulin from the surface coated liposomes achieved longer duration of oral hypoglycemic activity. Consequently, the surface coating should be the potential way to add desirable functions to the liposome for oral drug delivery^59^.

### Mucoadhesive polymeric systems:

Mucoadhesive polymeric systems are the most promising approach among several approaches. Mucoadhesive properties can provide an intimate contact with the mucosa at the site of drug uptake preventing a presystemic metabolism of peptides on the way to the absorption membrane in the gastrointestinal tract. Additionally, the residence time of the delivery system at the site of drug absorption is increased. Thus, we can achieve site-specific drug delivery by the use of mucoadhesive polymeric system. Mucoadhesive polymers are able to adhere to the mucin layer on the mucosal epithelium and thus results in the increase of oral drug bioavailability of protein and peptide drugs. These polymers decrease the drug clearance rate from the absorption site, thereby increasing the time available for absorption^15^.

Most of the current synthetic bioadhesive polymers are either polyacrylic acid or cellulose derivatives. Examples of polyacrylic acid-based polymers are carbopol, polycarbophil, polyacrylic acid (PAAc), polyacrylate, poly (methylvinylether-co-methacrylic acid), poly (2-hydroxyethyl methacrylate), poly(methacrylate), poly(alkylcyanoacrylate), poly(isohexylcyanoacrylate) and poly(isobutylcyanoacrylate). Cellulose derivatives include carboxymethyl cellulose, hydroxyethyl cellulose, hydroxypropyl cellulose, sodium carboxymethyl cellulose, methylcellulose, and methylhydroxyethyl cellulose. In addition, seminatural bioadhesive polymers include chitosan and various gums such as guar, xanthan, poly(vinylpyrrolidone), and poly(vinyl alcohol).

A new gastrointestinal mucoadhesive patch system (GI-MAPS) has been designed for the oral delivery of protein drugs^60^. The system consists of four layered films contained in an enteric capsule. The backing layer is made of a water-insoluble polymer, ethyl cellulose (EC). The surface layer is made of an enteric pH-sensitive polymer such as hydroxypropylmethylcellulose phthalate, Eudragit L100 or S100 and was coated with an adhesive layer. The middle layer, drug-containing layer, made of cellulose membrane is attached to the EC backing layer by a heating press method. Both drug and pharmaceutical additives including an organic acid, citric acid, and a non-ionic surfactant, polyoxyethylated castor oil derivative were formulated in the middle layer. The surface layer was attached to the middle layer by an adhesive layer made of carboxyvinyl polymer. After oral administration, the surface layer dissolves at the targeted intestinal site and adheres to the small intestinal wall, where a closed space is created on the target site of the gastrointestinal mucosa by adhering to the mucosal membrane. As a result, both the drug and the absorption enhancer coexist in the closed space and a high-concentration gradient is formed between inside the system and the enterocytes, which contributes to the enhanced absorption of proteins because most drugs are absorbed by a passive-diffusion mechanism. As a result, the absorption enhancer makes full use of its capacity. As the GI-MAPS is a novel drug-delivery system preparation, the fabrication method is the second hurdle to overcome in the launch of an oral preparation of proteins. However, recent advances in microfabrication technology in the semiconductor industry have made it possible to produce many micron-size GI-MAPS. Several approaches to produce the micron-size GI-MAPS are described and the future of these technologies is discussed.

Carbopol polymers have been shown to inhibit luminal degradation of insulin, calcitonin, and insulin-like growth factor-I (IGF-I) by trypsin and chymotrypsin^61^. Anionic polymers feature mucoadhesive properties via hydrogen bonding, van der Waal’s interactions and chain entanglement with the mucus^62^ forces stronger than the electrical repulsion caused by electrostatic interactions. In contrast, cationic polymers adhere to the negatively charged mucus mainly due to electrostatic forces^63^. As both anionic and cationic mucoadhesive polymers exhibit a high buffer capacity, a demanded microclimate regarding the pH can be adjusted and maintained over numerous hours within the polymeric network^64^.

On the contrary, the strong mucoadhesive properties of thiomers are believed to be based on additional covalent bonds between thiol groups of the thiomer and cysteine-rich subdomains of mucus glycoproteins^65^. This theory was confirmed by findings of mucoadhesion studies, where a higher amount of thiol groups on the polymer resulted in higher mucoadhesive properties^66^–^68^.

Although thiomers show strongly improved mucoadhesive properties, the adhesion of delivery systems being based on such polymers is nevertheless limited by the natural mucus turnover. The mucus turnover in the human intestine, for instance, was determined to be in the range of 12-24 h^69^. Consequently, at least within this time period, the adhesion of the delivery system will fail.

Hussain *et al*.^70^ have showed that surface conjugation of the bioadhesive molecule -tomato lectin increases the uptake of orally administered inert nanoparticles in rats. Improved intestinal absorption of 9-desglycinamide, 8-arginine vasopressin (DGAVP) was observed in rats *in vitro* as well as *in vivo* using the weakly cross-linked poly(acrylate) derivative polycarbophil dispersed in physiological saline (Haas and Lehr)^71^. Similarly, enhanced oral bioavailability of peptide and protein drugs was seen when these compounds were formulated with chitosan-EDTA conjugates^72^. The authors suggested that chitosan-EDTA conjugates protect peptide and protein drugs from enzymatic degradation across the GI tract.

## CONCLUSION

In conclusion, Delivering proteins and peptides by the oral route is extremely challenging. The very nature of digestive system is designed to breakdown these polypeptides into amino acids prior to absorption. The low bioavailability of drugs remains to be an active area of research. Several sites in the GIT have been investigated by researchers, but no major breakthrough with broad applicability to diverse proteins and peptides has been achieved. Considerable progress has been made over past few years in developing innovative technologies for promoting absorption across GI and numbers of these approaches are demonstrating potential in clinical studies. Chemical modification and use of mucoadhesive polymeric system for site-specific drug delivery seen to be promising candidates for protein and peptide drug delivery.

## References

[1] Rick S, Binghe W, Teruna S, Richard S (2005). Oral protein and peptide drug delivery. Drug delivery: Principles and applications.

[2] Adessi C, Sotto C (2002). Converting a peptide into a drug: Strategies to improve stability and bioavailability. Curr Med Chem.

[3] Adessi C, Sotto C (2004). Strategies to improve stability and bioavailability of peptide drugs. Frontiers Med Chem.

[4] Sayani AP, Chien YW (1996). Systemic delivery of peptides and proteins across absorptive mucosae. Crit Rev Ther Drug Carrier Syst.

[5] Torres LM, Peppas NA (2000). Transmucosal delivery systems for calcitonin: A review. Biomaterials.

[6] O' Hagan DT, Illum L (1990). Absorption of peptides and proteins from the respiratory tract and the potential for development of locally administered vaccine. Crit Rev Ther Drug Carrier Syst.

[7] Banga AK, Chien YW (1993). Hydrogel-based iontotherapeutic delivery devices for transdermal delivery of peptides-protein drugs. Pharm Res.

[8] Lee YC, Yalkowsky SH (1999). Effect of formulation on the systemic absorption of Insulin from enhancer free ocular devices. Int J Pharm.

[9] Burgess DJ, Pezznto JM, Johnson ME, Manasse HR (1993). Biotechnology and Pharmacy.

[10] Noha NS, Natalie DE, Alessio F (2006). Tight junction modulation and its relationship to drug delivery. Adv Drug Deliv Rev.

[11] Hoffman A, Ziv E (1996). Pharmacokinetic considerations of new insulin formulations and routes of administration. Drug Dispos.

[12] Saffran M, Kumar G, Savariar C, Burnham J, Williams F, Neckers D (1986). A new approach to the oral administration of insulin and other peptide drugs. Science.

[13] Fix JA (1996). Oral controlled release technology for peptides: Status and future prospects. Pharm Res.

[14] Vincent HL Lee, Satish DK, George MG, Werner R, Lee VH (1991). Oral route of protein and peptide drug delivery. Peptide and protein drug delivery.

[15] Ram IM, Ajit SN, Laura T, Duane DM (2003). Emerging trends in oral delivery of peptide and protein drugs. Crit Rev Ther Drug Carrier Syst.

[16] Kipnes M, Dandona P, Tripathy D, Still JG, Kosutic G (2003). Control of postprandial plasma glucose by an oral insulin product (HIM2) in patients with type 2 diabetes. Diabetes Care.

[17] http://www.nobexcorp.com.

[18] Hashimoto M, Takada K, Kiso Y, Muranishi S (1989). Synthesis of palmitoyl derivatives of insulin and their biological activities. Pharm Res.

[19] Brader ML, Sukumar M, Pekar AH, McClellan DS, Chance RE, Flora DB (2002). Hybrid insulin cocrystals for controlled release delivery. Nat Biotechnol.

[20] Bernkop SA (1998). The use of inhibitory agents to overcome the enzymatic barrier to perorally administered therapeutic peptides and proteins. J Control Release.

[21] Hutton DA, Pearson JP, Allen A, Foster SN (1990). Mucolysis of the colonic mucus barrier by faecal proteinases: Inhibition by interacting polyacrylate. Clin Sci.

[22] Lueben HL, de Leeuw BJ, Perard D, Lehr CM, de Boer AG, Verhoef JC (1996). Mucoadhesive polymers in peroral peptide drug delivery: I, Influence of mucoadhesive excipients on the proteolytic activity of intestinal enzymes. Eur J Pharm Sci.

[23] Andreas BS, Alexander HK, Verena ML, Thomas P (2004). Thiomers: potential excipients for non-invasive peptide delivery systems. Eur J Pharm Biopharm.

[24] Aungst B (2000). Intestinal permeation enhancers. J Pharm Sci.

[25] Lecluyse EL, Sutton SC (1997). *In vitro* models for selection of development candidates. Permeability studies to define mechanisms of absorption enhancement. Adv Drug Deliv Rev.

[26] Dong ZL, Lecluyse EL, Thakker DR (1999). Dodecylphosphocholine-mediated enhancement of paracellular permeability and cytotoxicity in Caco-2 cell monolayers. J Pharm Sci.

[27] Sood A, Panchagnula R (2001). Peroral route: An opportunity for protein and peptide drug delivery. Chem Rev.

[28] Hochman J, Artursson P (1994). Mechanisms of absorption enhancement and tight junction regulation. J Control Release.

[29] Sakai M, Imai T, Ohtake H, Otagiri M (1998). Cytotoxicity of absorption enhancers in Caco-2 cell monolayers. J Pharm Pharmacol.

[30] Haffejee N, Du Plessis J, Muller DG, Schultz C, Kotze AF, Goosen C (2001). Intranasal toxicity of selected absorption enhancers. Pharmazie.

[31] Obata Y, Sesumi T, Takayama K (2000). Evaluation of skin damage caused by percutaneous absorption enhancers using fractal analysis. J Pharm Sci.

[32] Tomita M, Hayashi M, Awazu S (1995). Absorption-enhancing mechanism of sodium caprate and decanoyl carnitine in Caco- 2 cells. J Pharmacol Exp Ther.

[33] Lindmark T, Kimura Y, Artursson P (1998). Absorption enhancement through intracellular regulation of tight junction permeability by medium chain fatty acids in Caco-2 cells. J Pharmacol Exp Ther.

[34] Fasano A, Uzzau S (1997). Modulation of intestinal tight junctions by Zonula Occludens toxin permits enteral administration of insulin and other macromolecules in an animal model. J Clin Invest.

[35] Karyekar CS, Fasano A, Raje S (2003). Zonula Occludens toxin increases the permeability of molecular weight markers and chemotherapeutic agents across the bovine brain microvessel endothelial cells. J Pharm Sci.

[36] Leone-Bay A, Sato M, Paton D, Hunt AH, Sarubbi D, Carozza M (2001). Oral delivery of biologically active parathyroid hormone. Pharm Res.

[37] Leone-Bay A, Ho KK, Agarwal R, Baughman RA, Chaudhary K, DeMorin F (1996). 4-[4-[(2-Hydroxybenzoyl)amino]phenyl]butyric acid as a novel oral delivery agent for recombinant human growth hormone. J Med Chem.

[38] http://www.emisphere.com.

[39] Milstein SJ, Leipold H, Sarubbi D, Leone-Bay A, Mlynek GM, Robinson JR (1998). Partially unfolded proteins efficiently penetrate cell membranes: Implications for oral drug delivery. J Control Release.

[40] Schatz G, Dobberstein B (1996). Common principles of protein translocation across membranes. Science.

[41] Wu SJ, Robinson JR (1999). Transport of human growth hormone across Caco-2 cells with novel delivery agents: Evidence for P-glycoprotein involvement. J Control Release.

[42] Kotze AF, de Leeuw BJ, Lueben HL, de Boer AG, Verhoef JC, Junginger HE (1997). Chitosans for enhanced delivery of therapeutic peptides across intestinal epithelia: *In vitro* evaluation in Caco-2 cell monolayers. Int J Pharm.

[43] Toorisaka E, Ono H, Arimori K, Kamiya N, Goto M (2003). Hypoglycemic effect of surfactant-coated insulin solubilized in a novel solid-in-oil-in-water (S/O/W) emulsion. Int J Pharm.

[44] Eiichi T, Masakazu H, Noriho K, Hiroshige O, Yuko K, Masahiro G (2005). An enteric-coated dry emulsion formulation for oral insulin delivery. J Control Release.

[45] Dollo G, Corrre PL, Guerin A, Chevanne F, Burgot JL, Leverge R (2003). Spray-dried redispersible oil-in-water emulsion to improve oral bioavailability of poorly soluble drugs. Eur J Pharm Sci.

[46] Christensen KL, Pedersen GP, Kristensen HG (2002). Physical stability of redispersible dry emulsions containing amorphous sucrose. Eur J Pharm Biopharm.

[47] Takeuchi H, Sasaki H, Niwa T, Hino T, Kawashima Y, Uesugi K (1992). Design of redispersible dry emulsion as an advanced dosage form of oily drug (vitamin E nicotinate) by spray-drying technique. Drug Develop Ind Pharm.

[48] Molina C, Cadorniga R (1995). Physical stability of lyophilized and sterilized emulsions. STP Pharma Pratiques.

[49] Myers SL, Shively ML (1992). Preparation and characterization of emulsifiable glasses: Oil-in-water and water-in-oil-in-water emulsions. J Colloid Interface Sci.

[50] Lowman AM, Morishita M, Kajita M, Nagai T, Peppas NA (1999). Oral delivery of insulin using pH-responsive complexation gels. J Pharm Sci.

[51] Kang SI, Bae YH (2002). pH-induced solubility transition of sulfonamide-based polymers. J Control Release.

[52] Kyriakides TR, Cheung CY, Murthy N, Bornstein P, Stayton PS, Homan AS (2002). pH sensitive polymers that enhance intracellular drug delivery *in vivo*. J Control Release.

[53] Sakuma S, Hayashi M, Akashi M (2001). Design of nanoparticles composed of graft copolymers for oral peptide delivery. Adv Drug Deliv Rev.

[54] Behrens I, Pena AT, Alonso MJ, Kissel T (2002). Comparative uptake studies of bioadhesive and non-bioadhesive nanoparticles in human intestinal cell lines and rats: The effect of mucus on particle adsorption and transport. Pharm Res.

[55] Carino GP, Jacob J, Mathiowitz E (2000). Nanosphere based oral insulin delivery. J Control Release.

[56] Patel HM, Túzél NS, Stevenson RW (1985). Intracellular digestion of saturated and unsaturated phospholipid liposomes by mucosal cells: Possible mechanism of transport of liposomally entrapped macromolecules across the isolated vascularly perfused rabbit ileum. Biochim Biophys Acta.

[57] Childers NK, Denys FR, McGee NF, Michalek SM (1990). Ultrastuctural study of liposome uptake by M cells of rat Peyer’s patch: An oral vaccine system for delivery of purified antigen. Reg Immunol.

[58] Tomizowal H, Aramaki V, Fujii Y (1993). Uptake of phosphotidylserine liposomes by rat Peyer’s patches following intraluminal administration. Pharm Res.

[59] Iwanaga K, Ono S, Narioka K, Morimoto K, Kakemi M, Yamashita S (1997). Oral delivery of insulin by using surface coating liposomes improvement of stability of insulin in GI tract. Int J Pharm.

[60] Eiamtrakarn S, Itoh Y, Kishimoto J, Yoshikawa Y, Shibata N, Murakami, Takada K (2002). Gastrointestinal mucoadhesive patch system (GI-MAPS) for oral administration of G-CSF, a model protein. Biomaterials.

[61] Bai JP, Chang LL, Guo JH (1996). Effects of polyacrylic polymers on the degradation of insulin and peptide drugs by chymotrypsin and trypsin. J Pharm Pharmacol.

[62] Gu JM, Robinson JR, Leung SH (1988). Binding of acrylic polymers to mucin/epithelial surface: Structure-property relationships. Crit Rev Ther Drug Carrier Syst.

[63] Woodley J (2001). Bioadhesion: New possibilities for drug administration?. Clin Pharmacokinet.

[64] Schnurch AB, Gilge B (2000). Anionic mucoadhesive polymers as auxiliary agents for the peroral administration of (poly)peptide drugs: Influence of the gastric fluid. Drug Develop Ind Pharm.

[65] Leitner VM, Walker GF, Schnurch AB (2003). Thiolated polymers: Evidence for the formation of disulphide bonds with mucus glycoproteins. Eur J Pharm Biopharm.

[66] Kast CE, Schnurch AB (2001). Thiolated polymers thiomers: Development and in vitro evaluation of chitosan-thioglycolic acid conjugates. Biomaterials.

[67] Roldo M, Hornof M, Caliceti P, Schnurch AB (2004). Mucoadhesive thiolated chitosans as platforms for oral controlled drug delivery: Synthesis and *in vitro* evaluation. Eur J Pharm Biopharm.

[68] Marschutz MK, Schnurch AB (2002). Thiolated polymers: Selfcrosslinking properties of thiolated 450 kDa poly(acrylic acid) and their influence on mucoadhesion. Eur J Pharm Sci.

[69] Schnurch AB, Krauland AH, Leitner VM, Palmberger T (2004). Thiomers: Potential excipients for non-invasive peptide delivery systems. Eur J Pharm Biopharm.

[70] Hussain N, Jani PU, Florence AT (1997). Enhanced oral uptake of tomato lectin-conjugated nanoparticles in the rat. Pharm Res.

[71] Haas J, Lehr CM (2002). Developments in the area of bioadhesive drug delivery systems. Exp Opin Biol Ther.

[72] Bernkop SA, Krajicek ME (1998). Mucoadhesive polymers as platforms for peroral peptide delivery and absorption: Synthesis and evaluation of different chitosan-EDTA conjugates. J Control Release.

